# Treatment patterns and appropriateness of antipsychotic prescriptions in patients with schizophrenia

**DOI:** 10.1038/s41598-021-92731-w

**Published:** 2021-06-29

**Authors:** Verónica Gamón, Isabel Hurtado, José Salazar-Fraile, Gabriel Sanfélix-Gimeno

**Affiliations:** 1 Health Services Research Unit, Fundación Para el Fomento de La Investigación Sanitaria y Biomédica de la Comunidad Valenciana, FISABIO (the Valencia Foundation for the Promotion of Health and Biomedical Research), Valencia, Spain; 2 Red de Investigación en Servicios de Salud en Enfermedades Crónicas (REDISSEC, ), Valencia, Spain; 3 Community Mental Health Centre Pere Bonfill, Valencia, Spain; 4 Consorcio Hospital General, Centro de Investigación Biomédica en Red de Salud Mental (CIBERSAM), Valencia, Spain

**Keywords:** Psychiatric disorders, Schizophrenia, Epidemiology, Comorbidities

## Abstract

Schizophrenia is a chronic mental condition presenting a wide range of symptoms. Although it has a low prevalence compared to other mental conditions, it has a negative impact on social and occupational functions. This study aimed to assess the appropriateness of antipsychotic medications administered to schizophrenic patients and describe current treatment patterns for schizophrenia. A retrospective cohort study was conducted in all patients over the age of 15 with an active diagnosis of schizophrenia and treated with antipsychotics between 2008 and 2013 in the Valencia region. A total of 19,718 patients were eligible for inclusion. The main outcome assessed was inappropriateness of the pharmacotherapeutic management, including polypharmacy use. Altogether, 30.4% of patients received antipsychotic polypharmacy, and 6.8% were prescribed three or more antipsychotics. Overdosage affected 318 individuals (1.6%), and 21.5% used concomitant psychotropics without an associated psychiatric diagnosis. Women and people with a comorbid condition like anxiety or depression were less likely to receive antipsychotic polypharmacy. In contrast, increased polypharmacy was associated with concomitant treatment with other psychoactive drugs, and only in user on maintenance therapy, with more visits to the mental health hospital. Overall, we observed a high level of inappropriateness in antipsychotic prescriptions. Greater adherence to guidelines could maximize the benefits of antipsychotic medications while minimizing risk of adverse effects.

## Introduction

Schizophrenia is a severe mental disorder affecting more than 21 million people worldwide. It is characterized by disordered thinking and behaviour; disorganized speech, perceptions, and emotions; and disturbances in self-awareness^1,2^. The aetiology of schizophrenia is still unknown, although a combination of genetic, environmental and psychosocial factors contributes to its development^3–8^. Although the prevalence of schizophrenia is lower compared to other psychoses (3.46 cases per year per 1000 inhabitants)^9^, it causes considerable impairment to personal, social, and professional functioning; is among the top 15 disability-causing diseases in the world^10^; and contributes to a decline in life expectancy^11–13^. Psychopharmacological treatment is aimed at reducing the frequency and severity of bouts, as well as improving people’s functionality and quality of life.

The recommendations proposed in clinical practice guidelines (CPGs) are followed and adapted according to patients’ needs and characteristics to favour therapeutic adherence. These guidelines recommend selecting the antipsychotic medication based on the phase of schizophrenia and considering whether treatment is for a relapse or due to a lack of therapeutic response. The treatment of choice would be second-generation antipsychotics (SGAs), as they produce fewer side effects than first-generation antipsychotics (FGAs). However, the selection should also be made considering the metabolic adverse effects of some SGAs^14–16^. In first episode psychosis, the dosing regimen should be the minimum effective dose, adjusted individually according to response and tolerability^17,18^. Maximum doses should never be exceeded due to increased risk of adverse effects, while combinations of more than one antipsychotic are generally only justified when gradually switching from one antipsychotic to another or when adding a drug to a long-acting injectable (LAI) antipsychotic regimen, to stabilize the disease^17,18^. The use of benzodiazepines, antidepressants, and mood stabilizers for treating comorbid conditions is considered acceptable^17,19^.

Information on the latter aspects of prescription patterns, such as antipsychotics polytherapy and dosing patterns, is scarce, an even more in large population-based cohorts from routine clinical practice. We aimed to assess the appropriateness of antipsychotic prescriptions, fundamentally based on polypharmacy and dosing patterns, describe other relevant aspects of the pharmacotherapeutic management patterns, and identify predictors of antipsychotic polypharmacy in all adults with schizophrenia from a complete European region covering 5 million inhabitants.

## Methods

### Study design

A retrospective cohort study was performed in all patients aged 15 and older with an active diagnosis of schizophrenia and treated with antipsychotics in the Valencia region from 2008 to 2013 within the Spanish National Health System.

### Setting

The study covered the population served by the Valencia Health Agency (VHA), about 97% of the 5 million inhabitants of the Valencia region, which is located on the Mediterranean coast of Spain.

### Population

The study includes all patients diagnosed with schizophrenia (International Classification of Diseases, ninth revision, Clinical Modification, ICD-9-CM, codes 295.0–295.9) in the electronic medical records of VHA between 1 January 2008 and 31 December 2013.

Inclusion criteria were schizophrenic patients treated with antipsychotic drugs, defined as at least one prescription/dispensation of an antipsychotic drug (Anatomical Therapeutic Chemical code N05A except N05AN) during the study period. The index date for the cohort was defined as the date of the first prescription/dispensation of one or more antipsychotics.

Exclusion criteria were patients with a first diagnosis of schizophrenia after the age of 65; people not registered in the municipal census, such as non-residents or temporary residents, people not covered by the Valencia Health System (VHS) (mainly Spanish government employees whose prescriptions are reimbursed by civil servant insurance mutual funds) and thus not included in the electronic records of the VHS.

### Data sources

The main data source was the Valencia health system integrated database (VID)^20^, which is the electronic health information system maintained by the Regional Ministry for Health in Valencia, linking individuals’ data from different databases through a unique patient identifier. The population information system registers sociodemographic data as well as dates and causes of VHA withdrawal, including death. The ambulatory information system provides information on active diagnoses, personal and family history, laboratory test results, and behavioural factors, among other data. The pharmaceutical module provides information on outpatient prescriptions and dispensations. The hospital discharge minimum basic data set is a synopsis of clinical and administrative information for all hospital admissions, including diagnoses and procedures. Finally, the accident & emergency department clinical record collects visits to this department and associated diagnoses, as well as the date of discharge.

### Inappropriateness indicators

The main outcome measure was the inappropriateness of the pharmacotherapeutic management in patients receiving an antipsychotic treatment for schizophrenia. For this purpose, we reviewed several CPGs^21–25^ and selected the most internationally well-known and/or the most recently published, namely, National Institute for Health and Care Excellence (NICE) Clinical Guidance^17^, the British Association for Psychopharmacology Schizophrenia Guidelines^18^ and the American Psychiatric Association Practice Guideline for the Treatment of Patient with Schizophrenia^19^ (Supplementary Table S1 summarizes CPG recommendations). Based on the recommendations in these CPGs and summaries of product characteristics (SmPC), we defined three indicators of inappropriateness: (1) concomitant use of more than one antipsychotic drug (polytherapy), defined as having more than one antipsychotic drug prescribed simultaneously within 30 days of the index date; (2) prescriptions exceeding the maximum allowable dose (MAD), defined as the upper limit of allowed medication dose that one may receive without the risk of significant side effects, as specified in the SmPC; and (3) simultaneous treatment with other psychotropic drugs without having an associated psychiatric diagnosis.

Additionally, other relevant aspects related to pharmacotherapeutic management patterns in patients with schizophrenia were assessed: type of antipsychotic drug prescribed (first-generation antipsychotic (FGA), second-generation antipsychotic (SGA), or combinations of the two), treatment with clozapine (alone or in combination with another antipsychotic drug), and prescriptions exceeding the maximum daily dose recommended by CPGs (MRDD), defined as the upper limit of the recommended daily dose above which the drug’s efficacy is not improved. Moreover, the most frequently prescribed concomitant psychotropic drugs were identified.

### Covariates

To describe the population, we collected demographic and clinical characteristics along with healthcare resource utilization in the 12 months preceding the index date. Comorbidity was defined as the presence of an active diagnosis for psychiatric comorbidities (Supplementary Table S2) on the electronic medical record within a 12-month period preceding the index date. The information during the previous year was used to define the use of concomitant medication and health services. Prescriptions of other psychotropic drugs within 15 days before and/or after the index date were also identified (Supplementary Table S3).

Covariates related to drug prescription or dispensation were: name of the medicinal product, pharmaceutical form, drug dose, dosage regimen, route of administration (oral or long-acting injectable), prescription/dispensing date and RELE (electronic dispensing) information.

### Statistical analysis

Sociodemographic and clinical characteristics of patients, as well as variables related to concomitant treatments and use of health system services, were described by means of descriptive analysis, using means and standard deviations (SD) for continuous variables and percentages for categorical variables. The cohort of patients treated with antipsychotics was divided by age (≤ 40 years vs ≥ 41 years) and according to whether they had received antipsychotic treatment in the year prior to the index date (prevalent vs new users).

Inappropriateness of pharmacotherapeutic management was assessed by proxy indicators: polypharmacy with different antipsychotic drugs (categorized by overlap of ≥ 2 or ≥ 3 antipsychotics), exceeding the maximum daily doses specified in the SmPC, and concomitant use of other psychotropic drugs and antipsychotics without an adequate psychiatric diagnosis.

In order to identify other relevant aspects of the management patterns of schizophrenic patients, other variables related to pharmacotherapeutic treatment were described: type of antipsychotic drug treatment, use of clozapine, and administration antipsychotics exceeding maximum recommended doses.

Sociodemographic, clinical and other variables associated with the concomitant prescription of two or more antipsychotics were identified and included in a multivariable logistic regression model using a stepwise approach. Odds ratios with their respective 95% confidence intervals were calculated, with entry and exit significance levels of 0.05 and 0.10, respectively. Three models were fitted: one for all patients and one each for new and prevalent users, to identify the covariates associated with polypharmacy use.

All statistical analyses were conducted using STATA 14R (StataCorp. 2015. *Stata Statistical Software: Release 14*. College Station, TX: StataCorp LP).

### Ethics approval

Because this is an observational, retrospective study, it does not entail any additional risk to patients nor does it affect new prescriptions of any medication. The protocol for this study was approved by the Ethics Committee for Clinical Research of the General Directorate of Public Health and the Centre of Public Health Research (CEIC DGSP-CSISP, July 31, 2015). All methods were performed in accordance with the relevant guidelines and regulations. The need for informed consent was waived by the aforementioned committee given the nature of the study. All patient data were anonymized and de-identified before being transferred to the research team for analysis, in compliance with Spanish laws on privacy (Act 15/1999) and patient’s rights (Act 41/2002).

## Results

**Figure 1:**
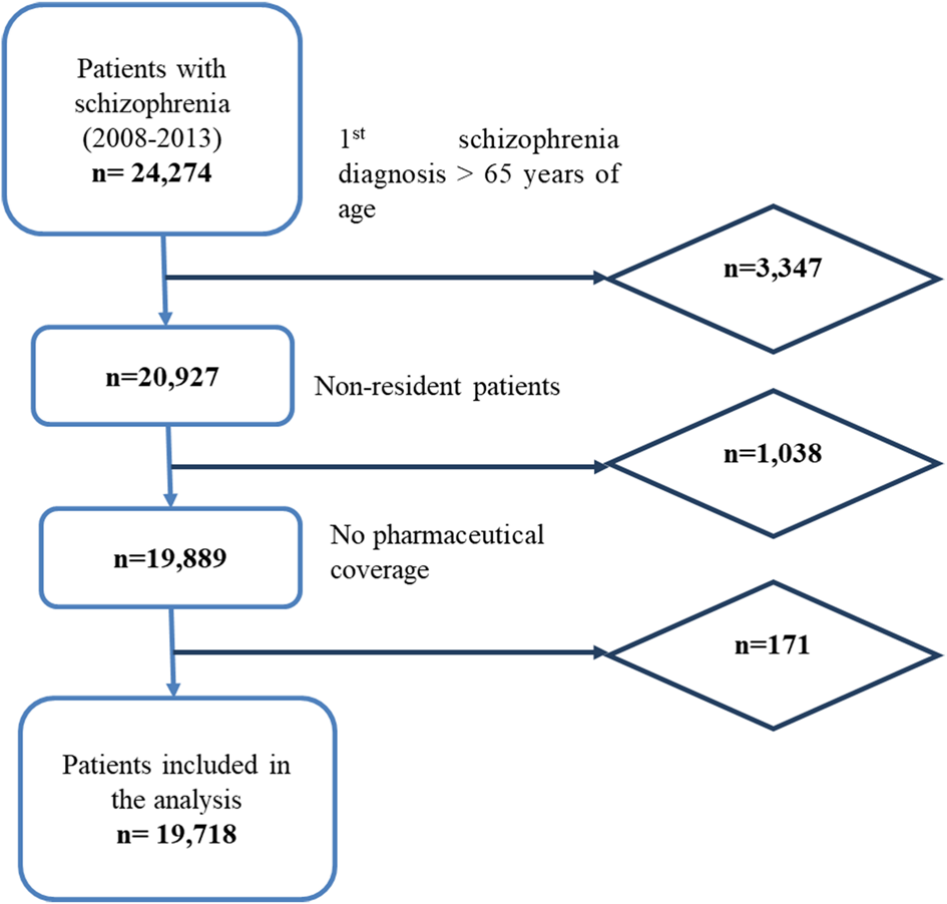
Study flow-chart.

The study included 19,718 patients (35.3% women) diagnosed with schizophrenia and at least one antipsychotic drug prescription (or dispensation) during the study period (Fig. 1). New users of antipsychotic medications comprised 41.3% (mean age 39.5, SD 12.1) of the sample, while the other 58.7% were prevalent users (mean age 41.6, SD 12.1); 52.2% were aged 15 to 40 years, and 47.8% were aged 41 years or more. Patient characteristics are described in Table 1.

**Table 1:** Patient characteristics.

	New users n = 8141 (41.3%)			Prevalent users n = 11,577 (58.7%)		
	16-40 years 4522 (55.6)	≥ 41 years 3619 (45.5)	Total 8141 (41.9)	16-40 years 5770 (49.8)	≥ 41 years 5807 (50.2)	Total 11,577 (58.7)
Female	1331 (29.4)	1517 (41.9)	2848 (35.0)	1683 (29.2)	2405 (41.4)	4088 (35.3)
**Psychiatric comorbidities**						
Depression	652 (14.4)	628 (17.4)	1,280 (15.7)	643 (11.1)	718 (12.4)	1,361 (11.4)
Dementia	22 (0.5)	60 (1.7)	82 (1.0)	82 (1.4)	112 (1.9)	194 (1.7)
Epilepsy	87 (1.9)	84 (2.3)	171 (2.1)	133 (2.3)	116 (2.0)	249 (2.2)
Parkinson’s disease	4 (0.1)	28 (0.8)	32 (0.4)	8 (0.1)	35 (0.6)	43 (0.4)
Anxiety	1310 (29.0)	869 (24.0)	2179 (26.8)	1257 (21.8)	1101 (19.0)	2358 (20.4)
Alcohol abuse	284 (6.3)	259 (7.2)	543 (6.7)	261 (4.5)	228 (3.9)	489 (4.2)
Drug abuse	796 (17.6)	216 (6.0)	1,012 (12.4)	629 (10.9)	145 (2.5)	774 (6.7)
Personality disorder	372 (8.2)	200 (5.5)	573 (7.0)	352 (6.1)	168 (2.9)	520 (4.5)
Sleep disorder	250 (5.5)	291 (8.0)	541 (6.7)	198 (3.4)	306 (5.3)	504 (4.4)
Delirium	82 (1.8)	52 (1.4)	134 (1.7)	75 (1.3)	59 (1.0)	134 (1.2)
Other disorders^a^	1234 (27.3)	645 (17.8)	1879 (23.1)	962 (16.7)	671 (11.6)	1633 (14.1)
**Concomitant psychotropic drugs**						
Antidepressants	781 (17.3)	765 (21.1)	1546 (19.0)	1690 (29.3)	1598 (27.5)	3288 (28.4)
Hypnotics/sedatives	484 (10.7)	487 (13.5)	971 (11.9)	547 (9.5)	773 (13.3)	1320 (11.4)
Antidementia drugs	2 (0.0)	39 (1.1)	41 (0.5)	13 (0.2)	41 (0.7)	54 (0.5)
Anxiolytics	1189 (26.3)	1165 (32.2)	2354 (28.9)	2528 (43.8)	2647 (45.6)	5175 (44.7)
Anticonvulsants	518 (11.5)	434 (12.0)	952 (11.7)	1566 (27.1)	1247 (21.5)	2813 (24.3)
Lithium	64 (1.4)	73 (2.0)	137 (1.7)	248 (4.3)	281 (4.8)	529 (4.6)
Antiparkinson drugs	140 (3.1)	167 (4.6)	307 (3.8)	1423 (24.7)	1599 (27.5)	3022 (26.1)
**Use of health services**						
Hospitalisations	1200 (26.5)	848 (23.4)	2,048 (25.2)	979 (17.0)	748 (12.9)	1727 (14.9)
Mental health hospital	992 (21.9)	552 (15.2)	1544 (18.9)	723 (12.5)	415 (7.1)	1138 (9.8)
No mental health hospital	280 (6.2)	344 (9.5)	624 (7.7)	317 (5.5)	384 (5.9)	701 (6.1
Mental health outpatient clinic	2549 (56.4)	1920 (53.1)	4469 (54.9)	5109 (88.5)	5018 (86.4)	10,127 (87.5)
No mental health outpatient cinic	3233 (71.5)	2403 (66.4)	5,636 (69.2)	4576 (79.3)	4654 (80.1)	9230 (79.7)
A&E	1832 (40.5)	1131 (31.2)	2963 (36.4)	298 (5.2)	228 (3.9)	526 (4.6)

^a^Other include: other organic psychosis, bipolar disorder, sexual disorder and acute stress reaction.

Regarding inappropriateness of schizophrenia treatment (Table 2), 26.4% of new users and 33.3% of prevalent users presented polypharmacy, receiving two or more antipsychotics, while 5.5% of new users and 7.7% of prevalent users were treated with at least three antipsychotics. As for antipsychotic dosage, the maximum licensed daily dose was exceeded in 107 new users (1.3%) and 211 people (1.8%) in maintenance therapy. A total of 9479 patients received concomitant treatment with psychotropic drugs, approximately half of whom did not have an associated psychiatric diagnosis (17.2% of new users and 24.5% of prevalent users).

**Table 2:** Inappropriateness of schizophrenia treatment.

	New users n = 8141 (41.3%)			Prevalent users n = 11,577 (58.7%)		
	16–40 years	≥ 41 years	Total	16–40 years	≥ 41 years	Total
**Polytherapy**						
≥ 2 AP	1230 (27.2)	918 (25.4)	2148 (26.4)	1956 (33.9)	1899 (32.7)	3855 (33.3)
≥ 3 AP	256 (5.7)	193 (5.3)	449 (5.5)	451 (7.8)	441 (7.6)	1341 (6.8)
**AP dosage**						
Exceeding MAD	58 (1.3)	49 (1.3)	107 (1.3)	87 (1.5)	124 (2.1)	211 (1.8)
**Concomitant psychotropic drugs**						
Without psychiatric dx	672 (14.8)	729 (20.1)	1401 (17.2)	1315 (22.8)	1526 (26.3)	2841 (24.5)

*AP* antipsychotic, *MAD* maximum allowable dose, *dx* diagnosis.

Table 3 shows patterns of pharmacotherapeutic management in schizophrenic patients. The vast majority of patients in monotherapy were treated with an SGA. Both new and prevalent users under treatment with two or more antipsychotics usually received at least one SGA (combination SGA + FGA or ≥ 2 SGAs). Only 4.5% of new users and 8.5% of prevalent users received combinations of two or more FGAs. Clozapine was prescribed in 189 (2.3%) new users, one third of whom were also receiving another antipsychotic treatment. Among prevalent users on clozapine, half took it alone, and the other half in combination with another antipsychotic. As for recommended daily doses, 194 new users (2.4%) and 371 prevalent users (3.2%) received doses of antipsychotics above the recommended range.

**Table 3:** Patterns of management in schizophrenic patients.

	New users n = 8141 (41.3%)			Prevalent users n = 11,577 (58.7%)		
	16–40 years	≥ 41 years	Total	16–40 years	≥ 41 years	Total
**Monotherapy**						
FGA	366 (11.1)	699 (25.9)	1065 (17.8)	440 (11.5)	958 (24.5)	1398 (18.1)
SGA	2926 (88.9)	2002 (74.1)	4928 (82.2)	3374 (88.5)	2950 (75.5)	6324 (81.9)
**Polytherapy**						
FGA (2 or more)	31 (2.5)	66 (7.2)	97 (4.5)	100 (5.1)	226 (11.9)	326 (8.5)
SGA (2 or more)	770 (62.6)	423 (46.1)	1193 (55.5)	1020 (52.2)	683 (36.0)	1703 (44.2)
FGA + SGA	429 (34.9)	429 (46.7)	858 (39.9)	836 (42.7)	990 (52.1)	1826 (47.4)
**Concomitant psychotropic drugs**						
	2039 (45.1)	1786 (49.4)	3825 (47.0)	2720 (47.1)	2934 (50.5)	5654 (48.8)
Use of clozapine	123 (2.7)	66 (1.8)	189 (2.3)	256 (4.4)	138 (2.4)	394 (3.4)
CLZ monotherapy	83 (2.5)	41 (1.5)	124 (2.1)	127 (3.3)	67 (1.7)	194 (2.5)
Clozapine + AP	40 (3.3)	25 (2.7)	65 (3.0)	129 (6.6)	71 (3.7)	200 (5.2)
**Starting dose AP**						
Exceeding MRDD	102 (2.2)	92 (2.5)	194 (2.4)	147 (2.5)	224 (3.9)	371 (3.2)

*FGA* first-generation antipsychotic, *SGA* second-generation antipsychotic, *CLZ* clozapine, *AP* antipsychotic, *MRDD* maximum recommended daily doses.

The prescription rate of combined antipsychotic with psychotropic drugs was similar among new versus prevalent users and between age groups (Supplementary Table S4). In patients given combined polypharmacy with other psychotropic drugs, the association most commonly prescribed was two antipsychotics and one psychotropic drug. A small percentage of people were treated with three antipsychotics plus one psychotropic drug. Moreover, most hypnotics and about 70% of antidepressants and anxiolytics were prescribed without any previous diagnosis of the related disorder (Supplementary Table S5).

**Figure 2:**
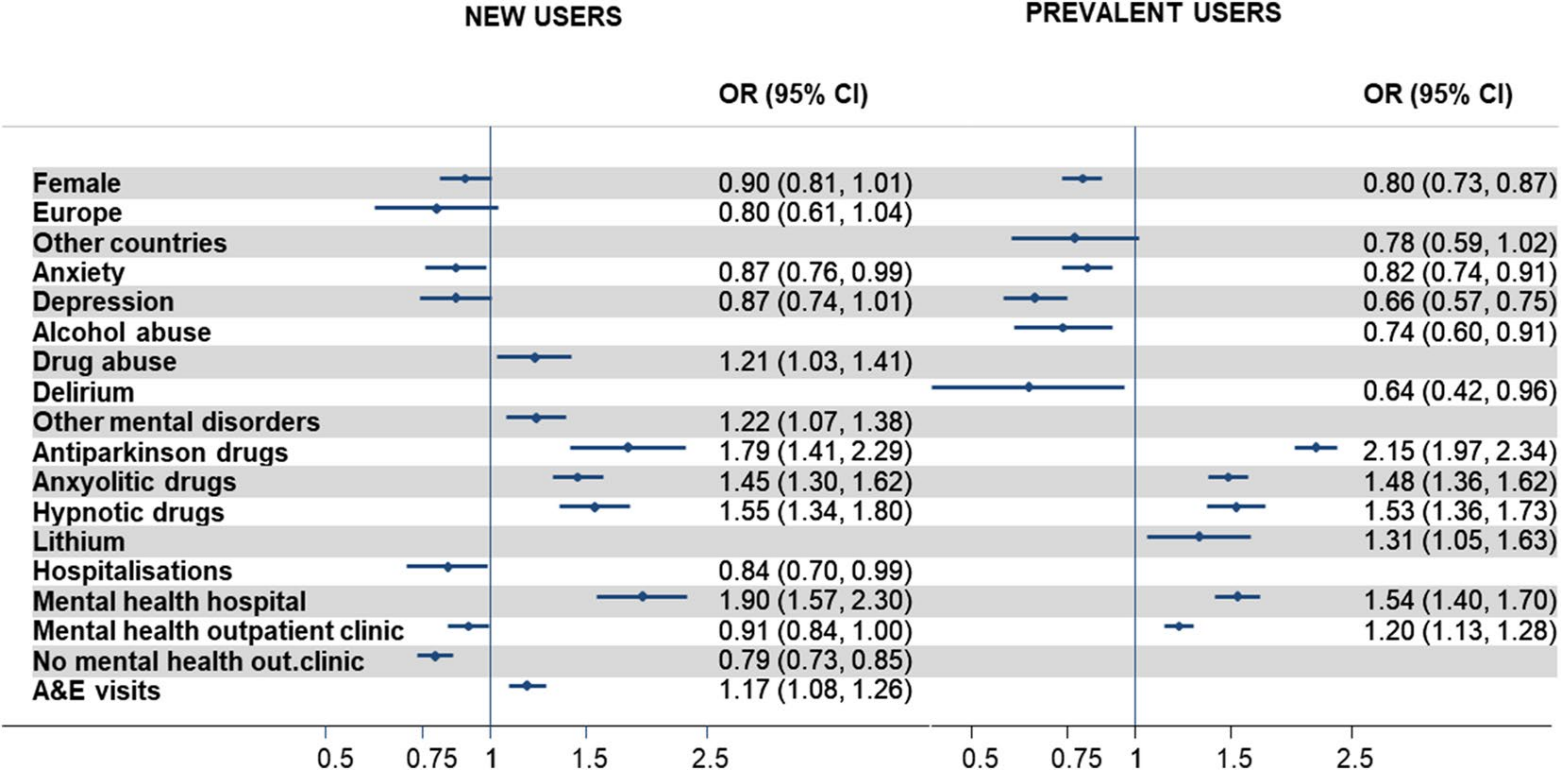
Factors associated with antipsychotic polypharmacy. Both models were adjusted for all variables included in Table 1 using backward-stepwise logistic regressions (with a removing probability of 0.10 and an entry probability of 0.05) to retain the significant variables. Covariates are not presented or cells are left empty in case of non-significance in the particular model. *OR* Odds Ratio, *out*. outpatient, *A&E* Accident and Emergencies, *CI* Confidence Interval.

Factors associated with the prevalence of antipsychotic polypharmacy are shown in Fig. 2. Women and people diagnosed with other psychiatric conditions, such as anxiety and depression, were less likely to receive polypharmacy. The concomitant use of psychotropic drugs, such as antiparkinsonians, anxiolytics, hypnotics, and lithium (the last one only in prevalent users) and visits to a mental health hospital were related to a 1.5- to 2.0-fold higher risk of being treated with more than one antipsychotic drug.

In new users, drug abuse, the presence of other mental disorders, and emergency visits increased the risk of antipsychotic polypharmacy by about 20%. In contrast, hospitalizations for any reason and visits to outpatient clinic and mental health outpatient clinic decreased the likelihood of antipsychotic polypharmacy. In prevalent users, alcohol abuse and delirium were associated with a lower risk of antipsychotic polypharmacy (OR 0.74; 95% CI 0.60; 0.91 and OR 0.64; 95% CI 0.42; 0.96, respectively). Unlike new users, prevalent users who visited mental health outpatient clinics more often were at increased risk of antipsychotic polypharmacy.

## Discussion

This retrospective study provides information on the inappropriateness of antipsychotic treatment and management patterns in a large population of patients with schizophrenia in a Mediterranean region of Europe. Our results show a high level of inappropriateness in antipsychotic prescriptions, with a quarter to a third of the schizophrenic patients under treatment with more than one antipsychotic drug.

A small but relevant percentage of patients exceeded MAD of antipsychotics specified in the SmPC. Approximately one in five people treated with concomitant psychotropic drugs did not have an associated psychiatric diagnosis. Other relevant aspects of antipsychotic treatment patterns were the high percentage of treatments with SGAs, both in monotherapy and in polytherapy, and the underuse of clozapine. In addition, the most common combination was two antipsychotics with one psychotropic, and most antidepressants, anxiolytics, and hypnotics were prescribed without an associated diagnosis. Finally, factors associated with increased polypharmacy use were concomitant treatment with other psychotropic drugs like anxiolytics and hypnotics as well as visits to the mental health hospital. On the contrary, women and those who were diagnosed with anxiety or depression were less likely to receive antipsychotic polypharmacy. These results were similar to the polypharmacy rate of 30.4% reported elsewhere^26,27^. About one out of every four new users was prescribed more than one antipsychotic, and 5.5% were prescribed three or more. This elevated rate of polypharmacy is potentially inappropriate given the lack of robust evidence associating polypharmacy with a higher response^28^ as well as the numerous studies asserting that antipsychotic polypharmacy is linked to increased adverse effects, poor medication adherence, and increased risk of drug interactions^29–31^. Prevalence of polypharmacy was higher in prevalent users: a third of these patients were prescribed two or more antipsychotic drugs, and 7.7% were prescribed three or more. These data coincide with several studies showing that antipsychotic polypharmacy becomes more prevalent as schizophrenia progresses^32,33^. Current CPGs recommend avoiding antipsychotic combinations except in specific circumstances (e.g. switching medications) and for limited time periods. However, these guidelines may be due for an update, as recent evidence suggests that polypharmacy may be better than monotherapy in certain clinical cases or for maintenance treatment^27,34,35^.

Regarding antipsychotic dosage, most prescriptions adhered to guidelines; however, a relevant percentage (1.6%) of people exceeded the maximum dose licensed in the SmPC, including 107 new and 211 prevalent users. Such overdosage increases the risk of toxicity, especially in polypharmacy, although more research is needed to estimate the clinical impact of these inappropriate treatment patterns on the cohort studied^36–38^. In addition, almost 3% of people in both patient groups received antipsychotic doses above the higher end of the recommended range. According to various studies, patients with schizophrenia who do not respond to treatment are prescribed high-dose therapy^39^. However, there is no evidence supporting this strategy, and two of the three CPGs reviewed in this study recommend starting antipsychotic treatment with the lowest effective dose and gradually moving to higher doses based on the patient's response. By contrast, antipsychotic doses above the highest dose thresholds are associated with higher rates of adverse effects, deterioration of cognitive function and an increased risk of mortality^30,31,40,41^.

As expected, the most frequent comorbidities were anxiety and depression^42^. Nearly half the included patients received concomitant treatment with psychotropic drugs, most frequently antidepressants, followed by anxiolytics. Although CPGs recommend benzodiazepines for anxiety and insomnia, especially in the acute phase of schizophrenia^43^, the overwhelming majority of the people receiving a hypnotic treatment did not have a diagnosis of sleep disorder, and a third of those who were prescribed an anxiolytic drug did not have a diagnosis of anxiety. As for depression, a common disorder in schizophrenia^44^, 70% of those treated with antidepressants did not have an associated diagnosis of depression. Although the use of these psychoactive drugs is accepted for comorbidities that are extremely prevalent in mental disorders, its use is not justified without the corresponding diagnosis. Notably, the use of anticonvulsants was more than twice as common in prevalent compared to new users, despite combining an antipsychotic with an antiepileptic is not supported by any firm evidence^45^ For antiparkinsonian drugs, this difference was even more pronounced (prevalent users 23.1% versus new users 3.8%), which can be attributed to the use of these drugs to reduce antipsychotic extrapyramidal side effects^46^.

With regard to the choice of antipsychotic medication, our results were in line with CPG recommendations, suggesting that the selection was primarily based on tolerance rather than efficacy. In more than 80% of the people on antipsychotic monotherapy, SGAs were the first-line drug, possibly due to their rapid efficacy and lower incidence of side effects compared to FGAs^47,48^. In keeping with previous studies, FGAs were more commonly prescribed in older patients^49^: 1 out of 4 patients aged 41 years and older were prescribed FGA monotherapy, compared to just 1 out of 10 younger patients. In patients with antipsychotic polypharmacy, the most frequent combination included at least one SGA, with 44.7% receiving an SGA plus FGA, and 48.2% receiving two SGAs. On the contrary, the association of two FGAs was uncommon (4.5% in new users and 8.5% in prevalent users). Similarly to the monotherapy group, combinations including at least one FGA in patients over 40 years of age were more frequent.

Nearly 3% of the included patients were given clozapine, data very similar to those obtained in other studies^26^. This proportion reflects an underuse of this drug, as research has shown that clozapine is indicated in up to 30% of patients diagnosed with treatment-resistant schizophrenia^50–52^. Instead, these patients are more frequently treated with additional antipsychotics and higher doses ^53^. Overall, the prevalence of clozapine use was significantly higher in young patients (15–40 years) and in prevalent users. Strikingly, 123 patients aged 15 to 40 years and 66 people aged 41 years or older were given clozapine in new users, 124 of them as monotherapy and 65 combined with another antipsychotic. Although the percentage is low (2.3%), all the reviewed CPGs agree that clozapine use should be restricted to patients with a clinically inadequate response after at least two trials with different antipsychotics, at an adequate dosage and duration^22^.

## Limitations

Reporting bias may exist due to the absence of data records or differences in data logging practices in electronic medical histories; however, this problem should always be taken into account when using data from routine clinical practice. Second, although currently prescriptions in the VHA are mostly electronic, 1% of prescriptions are still manual. The study included data from 2008, when this percentage was slightly higher, although still small (about 5%). In addition, in some electronic prescriptions the antipsychotic starting dose might have been handwritten, with only the maintenance dose registered in electronic health records. Third, overlap in prescribing two or more antipsychotics could exist when switching antipsychotic medications or formulations. We considered these cases to be polytherapy even though the patient was not taking both drugs simultaneously.

Fourth, due to the descriptive nature of the study, our results may not be extrapolated to other populations, representing the current therapeutic patterns of this population. Nevertheless, these results provide a broad in-depth overview of the pharmacotherapeutic management in routine clinical practice for a large, representative population of a complete region in Spain. Fifth, we have applied CPGs from the USA, and the UK to the population in the Valencia region, which has different characteristics from the populations where the guidelines were developed. Sixth, although the ideal design for an in-depth assessment of antipsychotic prescriptions appropriateness would probably be a prospective survey, the design used in the present study (population-based retrospective cohort study) offers some advantages as compared to prospective assessments. First, it includes all schizophrenic patients from a large European region (5 million inhabitants), which would be rather difficult and highly expensive in a prospective way, and thus, it allows providing evidence for the whole target population in routine clinical practice. And second, given that we have used a high quality and comprehensive electronic health system, reliability and completeness of the results provided is very high. Additionally, regarding maximum daily doses, we did this analysis for each medication individually, not taken into account when a patient was around the upper limit of two drugs, which would result in a high antipsychotic load even if, in both cases, they are below the limit, and this could potentially lead to higher probability of adverse events. And thus, we may be underestimating the real figures of overdosing. Finally, in patients with disease progression (prevalent users) treated with clozapine, it is difficult to know whether prescribing practices followed recommendations reserving the use of this antipsychotic drug for treatment-resistant schizophrenia. For this reason, we did not consider the use of clozapine as a main criterion of inappropriateness.

## Implications and future considerations

In summary, this study shows that despite CPG recommendations, antipsychotic polytherapy is still widespread in patients with schizophrenia. This practice, coupled with prescriptions for antipsychotic combinations plus other psychotropic drugs, may hinder therapeutic adherence and adequate management of schizophrenic patients due to increased side effects and drug interactions. In turn, this could have a negative impact on social integration and workforce reintegration. Further studies are needed to estimate the clinical impact arising from inappropriate antipsychotic treatment.

In the meantime, this study highlights the importance of mental health practitioners prescribing antipsychotics responsibly and rationally. In addition, we consider it crucial to address this type of disorders through a multidisciplinary team, achieving a balance between pharmacological and psychological therapy.

### Supplementary Information

Supplementary Information.
