# Author Correction: Treatment patterns and appropriateness of antipsychotic prescriptions in patients with schizophrenia

**DOI:** 10.1038/s41598-024-55913-w

**Published:** 2024-03-01

**Authors:** Verónica Gamón, Isabel Hurtado, José Salazar-Fraile, Gabriel Sanfélix-Gimeno

**Affiliations:** 1 Health Services Research Unit, Fundación Para el Fomento de La Investigación Sanitaria y Biomédica de la Comunidad Valenciana, FISABIO (the Valencia Foundation for the Promotion of Health and Biomedical Research), Valencia, Spain; 2 Red de Investigación en Servicios de Salud en Enfermedades Crónicas (REDISSEC, ), Valencia, Spain; 3 Community Mental Health Centre Pere Bonfill, Valencia, Spain; 4 Consorcio Hospital General, Centro de Investigación Biomédica en Red de Salud Mental (CIBERSAM), Valencia, Spain

Correction to: *Scientific Reports* 10.1038/s41598-021-92731-w, published online 29 June 2021

The Funding section in the original version of this Article was incomplete.

"This study was funded by Instituto de Salud Carlos III. Grant Number: PI17/02259."

now reads:

“This study was funded by Instituto de Salud Carlos III. Grant Number: PI17/02259, cofounded by the European Regional Development Fund (FEDER)."

The original Article has been corrected.

