# Supplementary material for: Treatment patterns and appropriateness of antipsychotic prescriptions in patients with schizophrenia
Source: Sci Rep. 2021 Jun 29;11:13509. doi: 10.1038/s41598-021-92731-w (PMC8241998; doi:10.1038/s41598-021-92731-w)
Supplement: Supplementary file 1 — Supplementary Information. [file 41598_2021_92731_MOESM1_ESM.pdf]

Treatment patterns and appropriateness of antipsychotic prescriptions in patients with schizophrenia

Authors Verónica Gamón, Isabel Hurtado, José Salazar-Fraile and Gabriel Sanfélix-Gimeno

Corresponding author: Isabel Hurtado

E-mail address: hurtado_isa@gva.es

SUPPLEMENTARY INFORMATION

Supplementary Table S1 CPGs recommendations for the pharmacological treatment of patients with schizophrenia

	NICE Clinical Guidance (2014)	BAP Guidelines (2017)	APA´s Practice Guideline (2020)
**General recommendations**			
AP prescription within the dosage range	✓	✓	
Do not use routinely combined AP	✓	✓	
Adjuncts to AP treatment can be: anticonvulsants, mood stabilisers, antidepressants and benzodiazepines (1)	✓	✓ (1)	✓
**First episode**			
Starting at the minimum dosage of AP	✓	✓	
No differences in efficacy of AP	✓	✓	✓
Do not use loading dose of AP	✓		✓
Continuing maintenance treatment and do not reduce dosage to reduce risk of relapse	✓	✓	✓
Use depot/LAI forms for relapse prevention	✓	✓	✓
Switch to another AP if there is no response after 6 weeks		✓	
**Acute episode (crisis)**			
SGA choice for first-line treatment	✓		
Use a minimum effective dose		✓	✓
Reassess medication dosage or switch of AP if no adequate response		✓	
**Maintenance treatment**			
Assess AP medication dosage, duration and adherence before switching to another AP		✓	
Avoid intermittent treatments	✓	✓	✓
Continuation of AP medication for at least 2 years		✓	
**Treatment-resistant schizophrenia (TRS)**			
Clozapine use when poor response after a trial with two different AP (at least a SGA)	✓	✓	✓
Clozapine augmentation with a second AP if inadequate reponse to clozapine	✓	✓	
Polypharmacy for relapse prevention			✓

Supplementary Table S2 Psychiatric comorbidities and corresponding CIE-9 codes

COMORBIDITY	CIE-9
**Depression**	296.2, 296.3, 296.5, 296.9, 300.4, 301.12
**Dementia**	290, 294, 330, 331
**Epilepsy**	345, 780.3, 780.33
**Parkinson´s disease**	332
**Anxiety**	300
**Alcohol abuse/dependence**	E860.0, 305.0, 571.0, 571.1, 571.2, 571.3, 357.5, 425.5, V11.3, 291, 303
**Drug abuse/dependence**	305.2, 305.3, 305.4, 305.5, 305.6, 305.7, 305.8, 305.9, 648.3, 292, 304
**Personality disorders**	301
**Sleep disorders**	347, 327, 307.4, 327.0, 327.2, 780.5
**Delirium**	293, 290.3, 291.0, 290.11, 290.41, 292.81
**Other mental disorders**	Other psychosis (297, 298, 299, 290.8, 290.9, 780.1), bipolar disorder (296.0, 296.1, 296.4,296.5, 296.6, 296.7, 296.8, 296.99) , sexual and gender identity disorder (302.x), acute stress reaction (308.1, 308.2, 308.3, 308.4, 308.9)

Supplementary Table S3 Concomitant treatment

CONCOMITANT TREATMENT	ATC
**Antidepressants**	N06AA, N06AB, N06AG, N06AX
**Anxyioltics**	N05BA, N05BB, N05BE, N05BX
**Hypnotics and sedatives**	N05C
**Anti-dementia drugs**	N06DA, N06DX
**Mood stabilisers**	N03AA, N03AE, N03AB, N03AF01, N03AF02, N03AX09, N03AX11, N03AX12
**Antiepileptic drugs**	N03A
**Lithium**	N05AN
**Antiparkinson drugs**	N04AA, N04BA, N04BC, N04BD, N04BX

Supplementary Table S4 Most frequent combinations of antipsychotic and psychotropic drugs

	New users n= 8.141 (41.3%)			Prevalent users n=11,577 (58.7%)		
	**16-40 years**	**>=41 years**	**Total**	**16-40 years**	**>=41 years**	**Total**
≥ **2 Antipsychotics**	1,230 (27.2)	918 (25.4)	2,148 (26.4)	1,956 (33.9)	1,899 (32.7)	3,855 (33.3)
+ 1 psychotropic drug	630(13.9)	508(14.0)	1,138(14.0)	1,098(19.0)	1,049(18.1)	2,147(18.5)
+ 2 psychotropic drugs	245(5.4)	219(6.1)	464(5.7)	438(7.6)	412(7.1)	850(7.3)
+ 3 psychotropic drugs	66(1.5)	76(2.1)	142(1.7)	117(2.0)	121(2.1)	238(2.1)
≥ **3 Antipsychotics**	256 (5.7)	193 (5.3)	449 (5.5)	451 (7.8)	441 (7.6)	892 (7.7)
+ 1 psychotropic drug	133(2.9)	113(3.1)	246(3.0)	283(4.9)	273(4.7)	556(4.8)
+ 2 psychotropic drugs	51(1.1)	46(1.3)	97(1.2)	123(2.1)	103(1.8)	226(2.0)
+ 3 psychotropic drugs	13(0.3)	12(0.3)	25(0.3)	45(0.8)	34(0.6)	79(0.7)

Supplementary table S5 Patterns of use of concomitant psychotropic drugs

	New users n= 8,141 (41.3%)			Prevalent users n=11,577 (58.7%)		
	**16-40 years**	**>=41 years**	**Total**	**16-40 years**	**>=41 years**	**Total**
**Antidepressants**						
≥1	821(9.5)	779(11.0)	1,600(10.1)	1,243(8.5)	1,204(11.3)	2,447(9.9)
≥1 without diagnosis	546(12.1)	476(13.2)	1,022(12.6)	974(16.9)	875(15.1)	1,849(16.0)
**Anxiolytics**						
≥1	1,441(31.9)	1,271(35.1)	2,712(33.3)	1,875(32.5)	2,094(36.1)	3,969(34.3)
≥1 without diagnosis	868(19.2)	814(22.5)	1,682(20.7)	1,336(23.2)	1,565(27.0)	2,901(25.1)
**Hypnotics/sedatives**						
≥1	484(10.7)	487(13.5)	971(11.9)	547(9.5)	773(13.3)	1,320(11.4)
≥1 without diagnosis	429(9.5)	397(11.0)	826(10.1)	492(8.5)	658(11.3)	1,150(9.9)
